# Classification and Genome-Wide Analysis of Chitin-Binding Proteins Gene Family in Pepper (*Capsicum annuum* L.) and Transcriptional Regulation to *Phytophthora capsici*, Abiotic Stresses and Hormonal Applications

**DOI:** 10.3390/ijms19082216

**Published:** 2018-07-29

**Authors:** Muhammad Ali, De-Xu Luo, Abid Khan, Saeed ul Haq, Wen-Xian Gai, Huai-Xia Zhang, Guo-Xin Cheng, Izhar Muhammad, Zhen-Hui Gong

**Affiliations:** 1 College of Horticulture, Northwest A&F University, Yangling 712100, China; alinhorti@yahoo.com (M.A.); abidagriculturist@gmail.com (A.K.); saeed_ulhaq@nwafu.edu.cn (S.u.H.); gaiwenxian@163.com (W.-X.G.); 2016060124@nwsuaf.edu.cn (H.-X.Z.); lvge2011@126.com (G.-X.C.); 2 Xuhuai Region Huaiyin Institute of Agricultural Sciences, Huaian 223001, China; loudex2002@163.com; 3 State Key Laboratory of Crop Stress Biology in Arid Areas, College of Life Sciences, Northwest A&F University, Yangling 712100, China; izeyaar@gmail.com

**Keywords:** chitin-binding protein, chitinase, pepper, expression, biotic stress, abiotic stress

## Abstract

Chitin-binding proteins are pathogenesis-related gene family, which play a key role in the defense response of plants. However, thus far, little is known about the chitin-binding family genes in pepper (*Capsicum annuum* L.). In current study, 16 putative chitin genes (CaChi) were retrieved from the latest pepper genome database, and were classified into four distinct classes (I, III, IV and VI) based on their sequence structure and domain architectures. Furthermore, the structure of gene, genome location, gene duplication and phylogenetic relationship were examined to clarify a comprehensive background of the CaChi genes in pepper. The tissue-specific expression analysis of the CaChi showed the highest transcript levels in seed followed by stem, flower, leaf and root, whereas the lowest transcript levels were noted in red-fruit. *Phytophthora capsici* post inoculation, most of the CaChi (*CaChiI3*, *CaChiIII1*, *CaChiIII2*, *CaChiIII4*, *CaChiIII6*, *CaChiIII7*, *CaChiIV1*, *CaChiVI1* and *CaChiVI2*) were induced by both strains (PC and HX-9). Under abiotic and exogenous hormonal treatments, the *CaChiIII2*, *CaChiIII7*, *CaChiVI1* and *CaChiVI2* were upregulated by abiotic stress, while *CaChiI1*, *CaChiIII7*, *CaChiIV1* and *CaChiIV2* responded to hormonal treatments. Furthermore, *CaChiIV1*-silenced plants display weakened defense by reducing (60%) root activity and increase susceptibility to NaCl stress. Gene ontology (GO) enrichment analysis revealed that CaChi genes primarily contribute in response to biotic, abiotic stresses and metabolic/catabolic process within the biological process category. These results exposed that CaChi genes are involved in defense response and signal transduction, suggesting their vital roles in growth regulation as well as response to stresses in pepper plant. In conclusion, these finding provide basic insights for functional validation of the CaChi genes in different biotic and abiotic stresses.

## 1. Introduction

Plants being sessile organisms are exposed to a number of stresses. External environmental fluctuations, different insect pest and pathogen considerably affect the growth, development, yield and quality [1]. To safe guard themselves against these threats, plants have evolved some sophisticated defense mechanisms. The inducible defense responses of plants include synthesis of signaling molecules, such as methyl jasmonate (MeJA), salicylic acid (SA) and ethylene (ET), which work in a complex network interaction that in turn regulates the expression of defense related genes (PR) and molecules such as reactive oxygen species (ROS), phytoalexins, proline, phenylpropanoids and pathogenesis-related genes [2,3]. Earlier studies revealed the significant role of these (PR) proteins in plant defense system [4,5]. During the biotic threat, plant defense mechanism consists of two typical interconnecting layers to develop plant immune system designated as effector-triggered immunity (ETI) and pattern-triggered immunity (PTI), thus participating in signal transduction [6,7]. A set of pathogenesis related (*PR-2* and *PR-5*) genes are involved in PTI and ETI, depending on the magnitude and time of the interacting signaling components [8,9].

Chitin-binding proteins (CBP), encoded by chitin-gene family, are PR proteins, which enhance resistance to different stresses in several crop plants [10,11,12,13,14]. These CBP proteins consist of one or several chitin-binding domains with high affinity and have a range of numerous complex glycoconjugates covering GlcNAc or *N*-acetyl-d-neuraminic acid (NeuNAc) as building blocks. Thus far, the chitinase responsible genes have been classified into seven different classes (classes I–VII) as they belongs to the glycoside_hydrolase_families, thus signifying that the chitinase isozymes were encoded by a family of multi-genes [4,15]. Some members of class I chitinases are localized in the vacuole, whereas other chitinases, such as the class III chitinases are positioned outside the cell [4]. Plant chitinases are responsible for the catalysis of chitin, the second most abundant polysaccharide after cellulose. Chitin is the part of the cell walls in most of fungi as well as in plants. Plant chitinases also have shown resistance to several pathogens, such as bacteria, viruses, and some abiotic stresses [16]. Certain chitinases are reported to take part in various physiological processes of plants, such as ethylene synthesis and embryogenesis [17]. CBPs are constitutively present in plant leaves, stems, seeds, flowers, and tubers. They are developmentally and tissue-specifically regulated [18,19]. Up to date, chitin genes have been cloned and characterized in numerous plants species, including *Arabidopsis thaliana* [20], *Triticum aestivum* [15], *Oryza sativa* [21], *Zea mays* [4] and *Sorghum bicolor* [22]. A class I chitin-binding proteins was isolated from *Hordeum vulgare* and has been shown antifungal activity [23]. The pathogen-inducible acidic class III chitinase proteins were isolated from *Nicotiana tobacum* after the infection of tobacco mosaic virus (TMV) [5] while endo-chitinase from *Trichoderma harzianum* showed higher resistance against phytopathogenic fungi in tobacco and apple [24,25].

Pepper (*Capsicum annuum* L.) is one of the essential Solanaceous vegetable crop possessing great economic value throughout the world. Its growth, yield and quality are reduced by numerous biotic factors such as bacterial wilt, *Phytophthora* blight, viral infections, insect pests and abiotic stresses (extreme temperatures, drought, salinity, and heavy metals) [26,27]. These stresses adversely affect the quality and yield of pepper plants. In response, plants have evolved some sophisticated defense mechanisms including oxidative burst and calluses into the cell wall and regulation of signaling networks to combat these stresses [7,28,29]. It has been reported that *Phytophthora capsici* infests pepper, eggplant, tomato, all cucurbits, and more recently snap and lima beans [30,31]. To control the attack of pathogen invasion in the host tissues, inducible biochemical reactions create a protective physiological condition [14].

The chitin-binding proteins are very important as they can enhance resistance against biotic and abiotic stress as well as in plant growth and development. The molecular function of chitin-binding protein genes in pepper plant are un-known. In the current study, sixteen chitin genes (CaChi) in pepper were mined through bioinformatics and their response to biotic and abiotic stresses and hormonal treatment were examined. Subsequently, the gene architecture, conserved domains, exon–intron structure, chromosomal location, gene duplication, gene ontology (GO) characterization, *cis-*acting regulatory elements in the promoter regions and phylogenetic relationships of the pepper chitin-binding protein were elucidated. This study provides a base for future research regarding pepper chitin-binding protein. Furthermore, differential expression was recorded against biotic (*Phytophthora capsici* two strains PC, HX-9) and abiotic (cold, drought, and salt) stress and hormonal treatment (SA, MeJA, and ABA) along with tissue specific expression in different plant parts. This study provides a foundation for further characterization of CaChi members in pepper and valuable information regarding function of this significant gene family in other important crops as well.

## 2. Results

### 2.1. Identification, Classification and Annotation of Chitin Genes in Pepper

**Figure 1:**
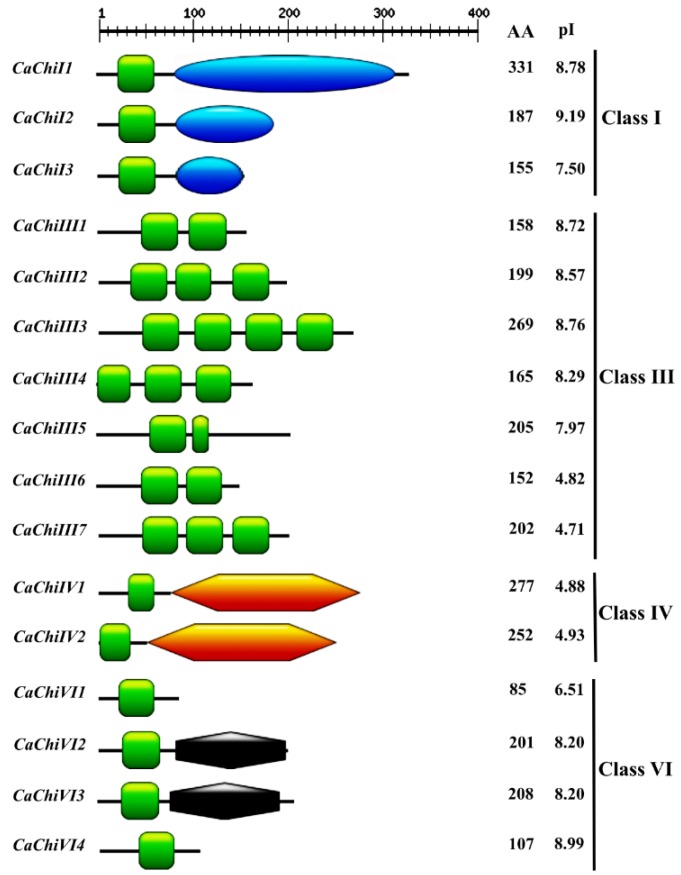
Domain architecture of CaChi classes I–VII in pepper and other plant species. The logos of domain organization were generated by Pfam database (Available online: http://pfam.xfam.org/search#tabview=tab0), and then further amendments were made with PhotoScape X. the aa: the number of amino acids; *p*I: isoelectric point; green 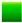: chitin binding domain (CBD); blue 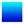: glycoside hydrolase 19 super family; orange 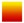: chitinase glycoside hydrolase 19; and 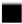: Barwin.

To comprehensively investigate and analyze the chitin-genes in pepper, the Hidden Markov Model (HMM) profile of the chitin-binding protein (Accession no. PF00187.17) was blast-searched in the pepper genome. As a result, 21 and 17 chitin genes were retrieved form CM334 and Zunla-1 databases, respectively. The gene sequences were aligned to avoid repetition and alternative splicing, and the longest sequences among them were chosen for further analysis. Among those 21 and 17 genes mined from the CM334 and Zunla databases, the genes having similar sequences with each other, were considered as a single gene. Consequently, we designed primer pairs (Table S1) for the amplification and confirmation of the doubtful gene sequences through cloning and sequencing. Finally, 16 predicted gene sequences were confirmed and then blast searched in NCBI. Nomenclature for the 16 CaChi was assigned based on their domains and chromosomal locations (Table 1 and Figure 1). The SMART results show that Chitin Binding Domain (CBD) was found in all 16 members while additional functional domains such as glycoside hydrolase_19_super family (*CaChiI1*, *CaChiI2*, and *CaChiI3*), chitinase glycoside_hydrolase_19 (*CaChiIV1* and *CaChiIV2*) and Barwin (*CaChiVI2* and *CaChi3*) were also found in this gene family (Figure 1 and Table S2). In addition, the characteristics of gene structure and protein size were quite different in CaChi gene family. The CDS of CaChi genes ranged from 258 bp (*CaChiVI1*) to 996 bp (*CaChiI1*), whereas the deduced proteins had 85–331 amino acids. The predicted *p*I values ranged from 4.71 (*CaChiIII7*) to 9.19 (*CaChiI2*), MW ranged from 9.06 (*CaChiVI1*) to 35.49 (*CaChiI1*) kDa and the instability index varied from 18.45 (*CaChiIV1*) to 68.89 (*CaChiIII4*) (Table 1 and Figure 1). The molecular formula shows that *CaChiIII3* contains the most (36) sulfur elements while *CaChiVI1* has the fewest (9) sulfur elements. All deduced proteins are shown in Table S2.

### 2.2. Construction of Phylogenetic Tree, Exon/Intron Structure and Conserved Motif Analysis

**Figure 2:**
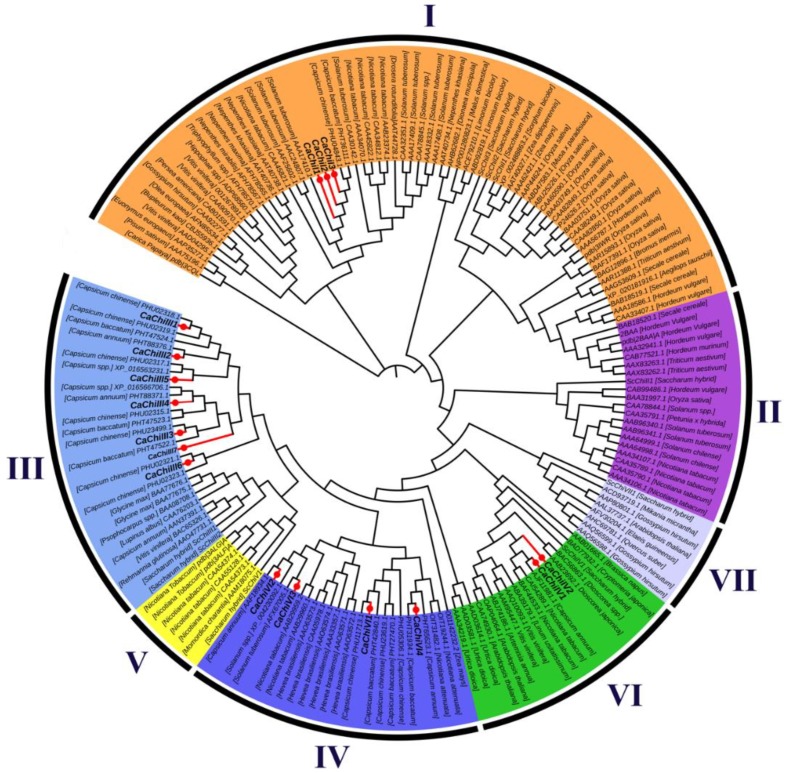
The phylogenic tree of chitin-binding protein family genes in pepper and other plant species. The phylogenetic tree was built using the neighbor-joining method and diagram was drawn using online iTOL (Available online: https://itol.embl.de/). The number of chitin-binding protein family genes were divided in I–VII well conserved groups.

To better understand the similarities and differences among the pepper and other plants chitin-binding protein genes, an unrooted phylogenetic tree was created using 162 chitin genes protein sequences from various plant species (Figure 2). These sequences used in the construction of phylogenetic tree were mainly from *Aegilops tauschii*, *Arabidopsis thaliana*, *Artemisia annua*, *Brassica napus*, *Brassica rapa*, *Bromus inermis*, *Bupleurum kaoi*, *Capsicum annuum*, *Capsicum baccatum*, *Capsicum chinense*, *Carica papaya*, *Cryptomeria japonica*, *Dionaea muscipula*, *Drosera rotundifolia*, *Euonymus europaeus*, *Gossypium barbadense*, *Gossypium hirsutum*, *Glycine max*, *Hevea brasiliensis*, *Hippophae rhamnoides*, *Hordeum vulgare*, *Limonium bicolor*, *Linum usitatissimum*, *Lupinus albus*, *Malus domestica*, *Mikania micrantha*, *Momordica charantia*, *Nepenthes khasiana*, *Nepenthes maxima*, *Nicotiana attenuate*, *Nicotiana benthamiana*, *Olea europaea*, *Oryza sativa*, *Persea americana*, *Pisum sativum*, *Psophocarpus tetragonolobus*, *Rehmannia glutinosa*, *Saccharum officinarum*, *Secale cereal*, *Sesamum indicum*, *Solanum tuberosum*, *Sorghum bicolor*, *Triphyophyllum peltatum*, *Triticum aestivum*, *Urtica dioica*, *Vitis vinifera*, *Zea diploperennis*, and *Zea mays.* The analysis shows that 16 CaChi were clearly classified into four distinct classes according to their sequence relatedness with previous research. Three CaChi (*CaChiI1*, *CaChiI2* and *CaChiI3*) were clustered in class I, seven CaChi (*CaChiIII1*, *CaChiIII2*, *CaCHiIII3*, *CaChiIII4*, *CaChiIII5*, *CaChiIII6* and *CaChiIII7*) in were clustered class III, two CaChi-genes (*CaChiIV1* and *CaChiIV2*) were clustered in class IV, and four CaChi (*CaChiVI1*, *CaChiVI2*, *CaChiVI3* and *CaChiVI4*) were clustered in class VI (Figure 2). Each class is highlighted with a different color following the previous chitin-binding protein genes classification [32,33].

**Figure 3:**
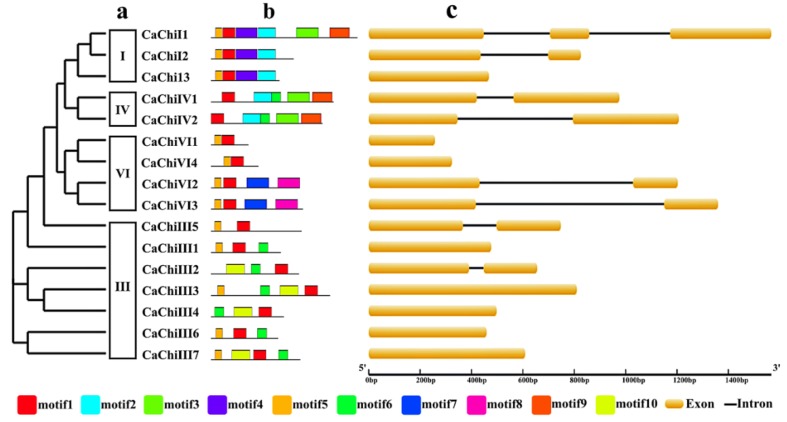
Phylogenetic relationship, domain organization and conserved motifs analysis of chitin-binding proteins family genes in pepper. (**a**) Phylogenetic analysis and classification of pepper genes. The phylogenetic tree was constructed via online iTOL (Available online: https://itol.embl.de/). (**b**) Motif analysis of pepper CaChi proteins. Motifs, numbered 1–10, were identified using MEME 4.11.2 software and are illustrated by different colors. Amino acid sequence of each motif is shown in Table S3. (**c**) Exon/intron structures of pepper chitins genes. Yellow boxes represent exons and introns are represented by black lines between two exons.

The members within certain class exhibited higher identity percentage of the amino acids sequences (Figure 3a). The exon/intron structure analysis showed that out of 16 CaChi, 8 CaChi (50%) had no introns while 8 CaChi (50%) contained only one intron (Figure 3c). The conserved motifs of CaChi proteins were identified by online MEME server (Available online: ). A sum of ten putative different motifs were obtained (Table S3). Motif 1 was found in all CaChi while motif 5 was found in most CaChi (except *CaChiIV1*, *CaChiIV2*, *CaChiIII2*, and *CaChiIII4*). Motif 6 was present in 50% of the CaChi (*CaChiIII1*, *CaChiIII2*, *CaChiIII3*, *CaChiIII4*, *CaChiIII6*, *CaChiIII7*, *CaChiIV1* and *CaChiIV2*). Motifs 2 and 10 were present in five and four CaChi, respectively. Motifs 3, 4 and 9 each were found in three different sequence of CaChi while motifs 7 and 8 each existed in two CaChi (Figure 3b and Table S3).

### 2.3. Chromosomal Location and Genes Duplication

**Figure 4:**
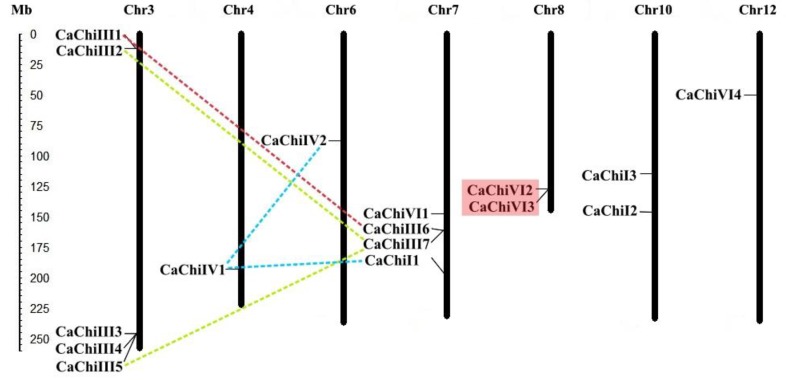
Chromosomal localization of CaChi of pepper plant, where the red shading box represents the tandem duplicated region. While the red, green and blue lines connection displaying segmentally duplicated genes.

According to the chromosomal location of the chitin-binding protein genes in pepper, the 16 CaChi were distributed across 7 out of 12 chromosomes of the pepper. Intriguingly, all CaChi members are random and non-randomly distributed across the chromosomes (Figure 4). The results showed that chromosome 3 had the highest number of CaChi (31.25%) as compared to other chromosomes. There were four genes (25%) on chromosome 7, while chromosomes 8 and 10 each have two genes. The remaining chromosomes (4, 6 and 12) each contained one gene. The duplication analysis showed that *CaChiIII1* have segmental duplication with *CaChiIII6* which occurred on chromosomes 3 and 7, respectively (Figure 4). *CaChiIII7* has two segmental duplication events with *CaChiIII2* and *CaChiIII5. CaChiIV1* also exhibited two segmental duplication events with *CaChiI1* and *CaChiIV2*. Moreover, one tandem duplication event was observed between *CaChiVI2* and *CaChiVI3*, which occurred on chromosome 8. Taken together, our findings suggest that, in the expansion of pepper CaChi genes, tandem and segmental duplication have an important contribution.

### 2.4. Cis-Acting Elements and Gene Ontology (GO) Analysis of CaChi

**Figure 5:**
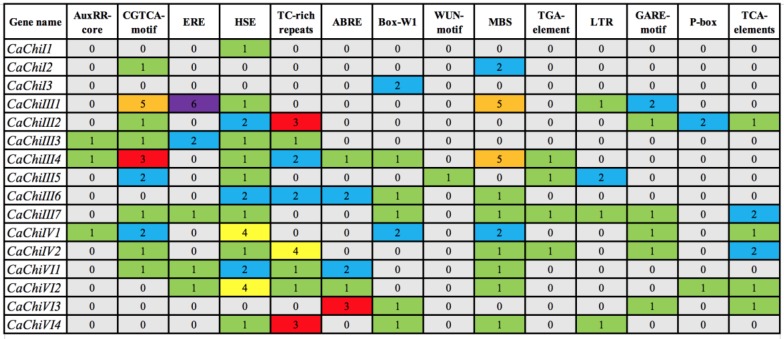
*Cis*-acting regulatory elements in the promoter regions of CaChi genes. The *cis*-element positions in the individual CaChi promoter region was inferred from the Plant CARE website (Available online: http://bioinformatics.psb.ugent.be/webtools/plantcare/html/). The different number of *cis* regulatory elements represent in different colors.

To examine the possible *cis*-acting elements involvement in the stimulation of defense-related genes, the 1.5 kb upstream region from the start codon of all the CaChi genes were analyzed with Plant CARE online server. The silico analysis revealed that *Cis-*elements conferring responsiveness to plant hormones, biotic and abiotic stresses were found in the promoters of the CaChi. As shown in Figure 5 and Table S4, the heat stress elements (HSE) were identified in the promoters of all CaChi (except *CaChiI2*, *CaChiI3* and *CaChiVI3*), in which the HSE in the promoters of the *CaChiIV1* and *CaChiVI2* were highest (4) followed by *CaChiIII2*, *CaChiIII6* and *CaChiIV1* (each have 2). MeJA-responsiveness elements (CGTCA-motif) were found in the promoter region of 10 CaChi, where *CaChiIII1* had the highest number (5) of elements followed by *CaChiIII4* (3), while *CaChiIII5* and *CaChiIV2* each have two elements. *Cis*-acting elements involved in abscisic acid (ABA) responsiveness elements (ABRE), salicylic acid responsiveness (TCA-element) and ethylene-responsive element (ERE) were found in the promoter regions of six CaChi. The MYB binding site involved in drought-inducibility (MBS), resistance and stress responsiveness (TC-rich repeats), GA-responsive element (GARE-motif) and fungal elicitor-responsive element (W-box) were found in the promotor regions of 10, 8, 6 and 7 CaChi genes, respectively. The *cis*-acting element involved in low temperature sensitivity (LTR) was found in the promoter region of four CaChi. In addition, GA-responsive element (P box) and auxin-responsive elements (TGA-element and AuxRR-core) were also found in some of the CaChi promoter regions. All of the anticipated *cis*-elements were involved in response to signaling molecules and stresses.

**Figure 6:**
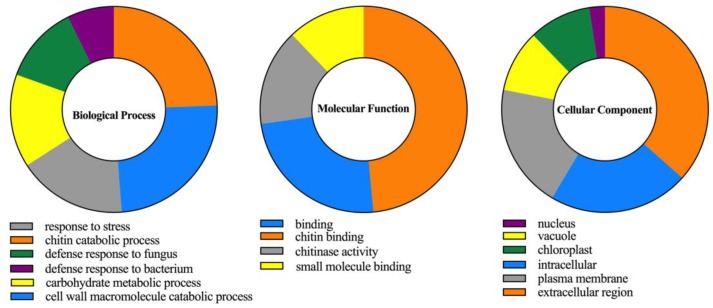
Gene ontology analysis of CaChi proteins in three categories (Biological processes, molecular functions and cellular component) using Blast2Go program. Different colors which are indicated near the graphics show different biological process, molecular functions and cellular component of pepper chitin-binding protein family genes.

Gene ontology (GO) enrichment analysis of CaChi were predicted by the gene ontology slim analysis using Blast2GO tool. The analysis comprised three categories, i.e., biological process, molecular function, and cellular component same as mentioned by Di et al. (2018) [34]. Our results showed that chitin catabolic processes and cell wall macromolecule catabolic processes, defense response to fungus, bacterium and response to stress were the highly regulated functions having role in biological process which support the function of the CaChi in the cell. In addition, prediction of molecular functions of CaChi proteins indicated that they had mostly involved in chitin binding capacity, chitinase activity and small molecules binding while cellular component analysis revealed that CaChi mostly localized in extracellular region. Furthermore, they can accumulate in subcellular parts of the cell such as vacuole, chloroplast, vacuole and plasma membrane (Figure 6).

### 2.5. Expression Analysis of CaChi under *Phytophthora capsici* Strains Inoculation 

**Figure 7:**
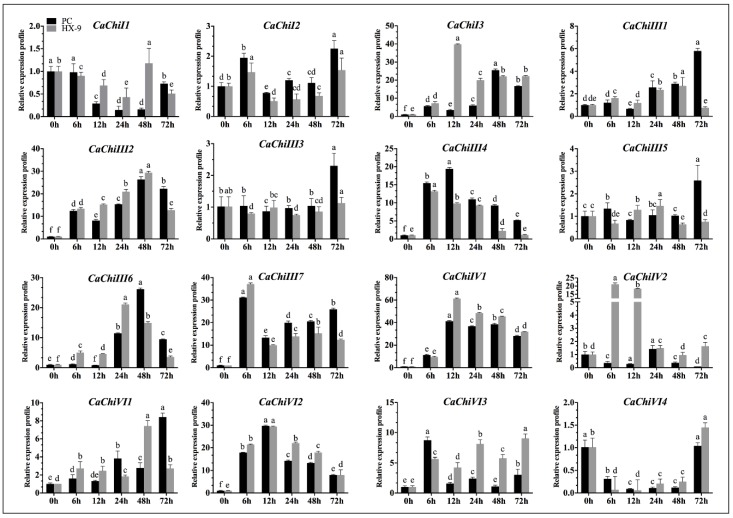
Expression profiles of CaChi in response to different strains of *Phytophthora capsici* (PC and HX-9). The samples were collected at different time points (0, 6, 12, 24, 48 and 72 hpt) and were analyzed by qRT-PCR. Mean values and SDs for three replicates are shown. Small letters (a–e) represent significant differences (*p* < 0.05).

To examine the transcription levels of CaChi against the virulent (HX-9) and avirulent (PC) strain of *Phytophthora capsici*, pepper plants were inoculated with *P. capsici* via root drenching and their expression levels were analyzed by qRT-PCR. The results exposed that, post *P. capsici* inoculation, the CaChi were differentially expressed (Figure 7). Twelve CaChi (75%) were upregulated on different time points, and three members (*CaChiI1*, *CaChiIII3* and *CaChiVI4*) (18.75%) were downregulated to both strains on maximum time points. Initially, *CaChiI1* and *CaChiVI4* exhibited downregulation after inoculation with virulent strain (HX-9), then *CaChiI1* upregulated on 48 hpi and *CaChiVI4* on 72 hpi, which were 1.18 and 1.45, respectively. However, *CaChiIII2***,** *CaChiIII6* and *CaChiVI1* exhibited progressive upregulation at all the time points in both strains, but *CaChiIII2* was reached to peak (29.39) at 48 hpi in HX-9, while *CaChiIII6* showed the highest transcription level after PC post inoculation (48 h), i.e., 26.17. Whereas *CaChiVI2* peaked at 12 hpi in virulent (29.38) and avirulent (29.75), *CaChiIV1* showed the highest expression compared with other CaChi, reaching a maximum at 38.52 (PC) and 45.51 (HX-9) and then slightly downregulated. Meanwhile, in the event of avirulent strain inoculation, *CaChiI2* (2.25), *CaChiIII1* (5.80) *CaChiIII3* (2.30) and *CaChiIII5* (2.59) were not predominantly upregulated but, at 72 hpi, showed slight expression. However, *CaChiIII4* and *CaChiVI2* were upregulated following the same pattern and reached a maximum 19.39 and 29.75, respectively, at 12 hpi. *CaChiI3* and *CaChiIV2* exhibited significant expression only to virulent (HX-9) strain. *CaChiIII7* revealed upregulation for both virulent and avirulent strains and reached a peak (31.10 and 37.13 respectively) at 6 hpi, then downregulated, and subsequently upregulated. Six hours post inoculation, *CaChiVI3* exhibited the highest transcription in PC strain and then later it was downregulated at every other time point; however, for HX-9 strain, its transcriptional level was raised.

### 2.6. Expression Profile of CaChi in Response to Abiotic Stresses

**Figure 8:**
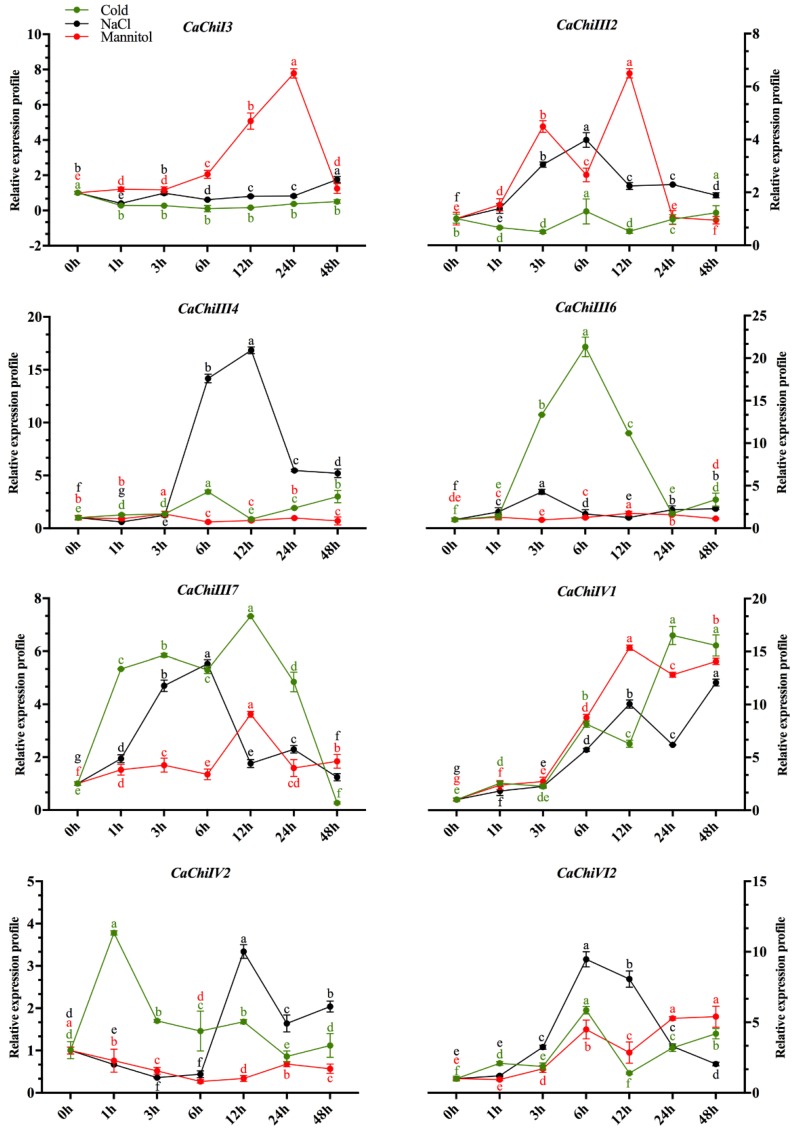
Expression profiles of CaChi genes in response abiotic stresses. The inducible expression patterns performed by qRT-PCR under Sodium chloride (NaCl) and Mannitol. Mean values and SDs for three replicates are shown. Small letters (a–e) represent significant differences (*p* < 0.05).

To examine the expression levels of the CaChi in response to abiotic stresses, eight representative genes (*CaChiI3*, *CaChiIII2*, *CaChiIII4*, *CaChiIII6*, *CaChiIII7*, *CaChiIV1*, *CaChiIV2* and *CaChiVI2*) were selected from the CaChi, in which at least one gene was selected from each class on the basis of their *cis-*acting elements response and expression to *P. capsici*. Then, they were subjected to NaCl, mannitol and cold stresses (Figure 8). *CaChiI3* showed no response to cold and NaCl stress while in response to mannitol it was gradually upregulated, reaching a maximum at 24 hpt (7.78), and then downregulated. *CaChiIII2* showed a slight upregulation at 6 hpt in response to NaCl while exhibited concomitant up- and downregulation in response of mannitol stress. In the case of cold stress, no expression was recorded. In response to NaCl, *CaChiIII4* initially exhibited no response, then upregulated at 6 hpt, reached a maximum at 12 hpt (16.84) and then showed a slight downregulation at 24 and 48 hpt, whereas no significant response was observed in response to cold and mannitol (Figure 8). *CaChiIII6* was not regulated by mannitol stress, whereas abrupt changes were observed to NaCl stress; however, highest expression was noticed at 6 hpt (21.32) in response to cold stress, where the expression was reduced in later hours. *CaChiIII7* was gradually upregulated in response to NaCl, reached a maximum at 6 hpt (5.53) and then downregulated. In response to mannitol, a slight up regulation was noted at 12 hpt and then downregulated similarly. In cold stress, significant expression (7.32) was observed in all time points. The transcript level of *CaChiIV1* was highly induced by mannitol, cold and NaCl at 12, 24 and 48 hpt, which were more than 12, 16 and 15 folds, respectively. *CaChiIV2* was initially downregulated by NaCl and mannitol stress and then exhibited an abrupt upregulation in response to NaCl at 12 hpt (3.34) and again downregulated. In response to mannitol stress, no significant expression occurred. In cold stress, it shown initial abrupt upregulation and then smoothly downregulated (Figure 8). Responding to NaCl stress, *CaChiVI2* was gradually upregulated, peaked at 6 hpt (9.47) then smoothly downregulated. In response to cold and mannitol, it was upregulated and reached a maximum at 6 hpt (5.86) and 48 hpt (5.40), respectively.

### 2.7. Expression Profile of CaChi in Response to Hormonal Treatments

**Figure 9:**
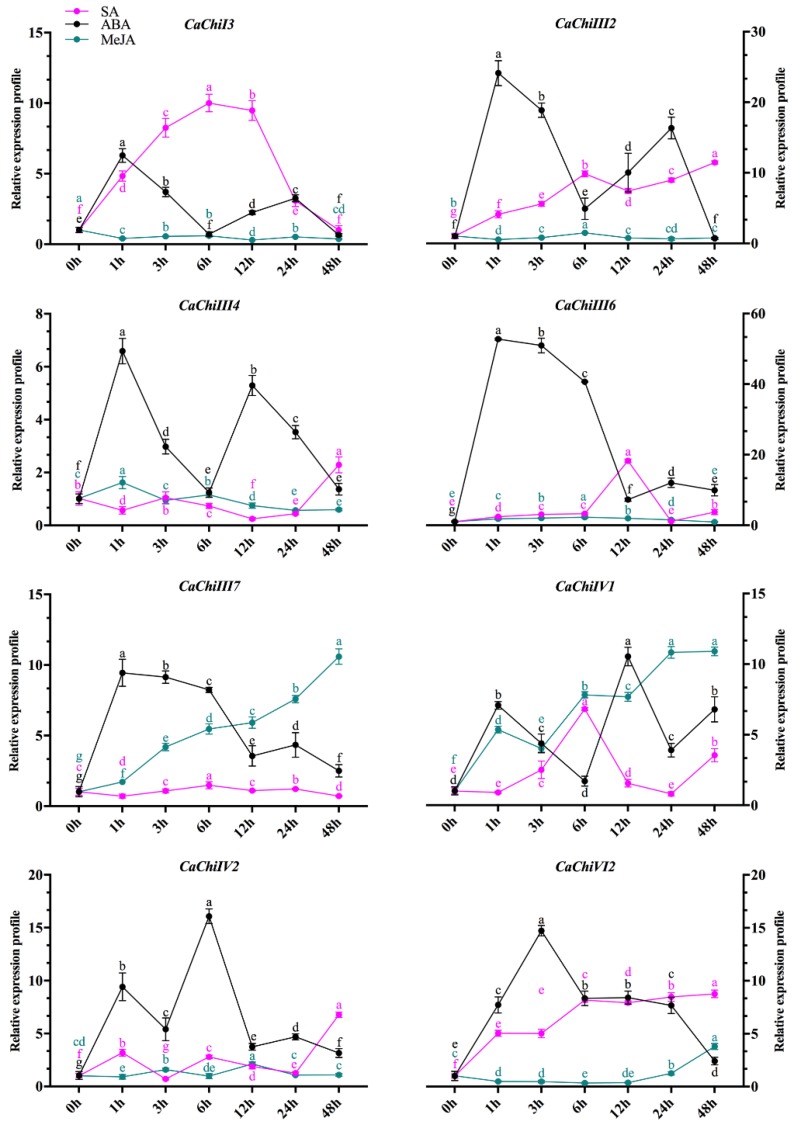
Expression profiles of CaChi in response hormones application. The inducible expression patterns performed by qRT-PCR under Salicylic acid (SA) and methyl-jasmonate (MeJA). Mean values and SDs for three replicates are shown. Small letters (a–f) represent significant differences (*p* < 0.05).

Phyto-hormones and plant signaling molecules, such as MeJA, SA and ABA, are involved in various stress signaling pathways [35,36,37,38]. The above selected eight CaChi genes were also exposed to exogenous hormonal (MeJA, SA and ABA) treatments to explore the response of these target genes. We investigated the expression profiles of CaChi-genes in AA3 leaves. As shown in Figure 9, post SA and ABA treatment, five CaChi (*CaChiI3*, *CaChiIII2*, *CaChiIII6*, *CaChiIV1* and *CaChiVI2*) were significantly upregulated (>10, 11, 18, 6, 8 folds against SA and >6, 24, 52, 10 and 14 folds against ABA, respectively) at different time points, while *CaChiIV1* and *CaChiIV2* were gradually upregulated over time and maximum expressions at 48 (10 folds) and 12 hpt (two folds) were recorded in response to MeJA treatment. The *CaChiIII4* was initially upregulated 1 hpt after ABA treatment (>5 folds), and then irregular changes were noticed, while, in response to MeJA and SA, the expression level was very low or even not obvious, except for SA where an abrupt upregulation (2.29) was noted at all given time points. *CaChiIII7* responded to MeJA and reached a peak at 48 hpt (10.60), whereas the response to ABA was antagonistic-like initially where an upregulation and then smooth decline were observed. Expression was not induced by SA treatment. The transcript levels of *CaChiIV1* gene were induced by MeJA, steadily increased and reached a peak (10.90) at 48 hpt. In the case of SA treatment, it was gradually upregulated, reached a peak (6.82) at 6 hpt, downregulated at 12 and 24 hpt, and again upregulated at 48 hpt. After ABA treatment, transcription level was high at 1 (7 folds) and 12 hpt (10 folds). The *CaChiIV2* was upregulated by SA, reached to peak at 48 hpt (8.76). After ABA application, it abruptly upregulated at 1 and 12 hpt (>9 and 16 folds, respectively), while it showed no significant response to MeJA.

### 2.8. Expression Patterns of CaChi-Genes in Different Tissues 

**Figure 10:**
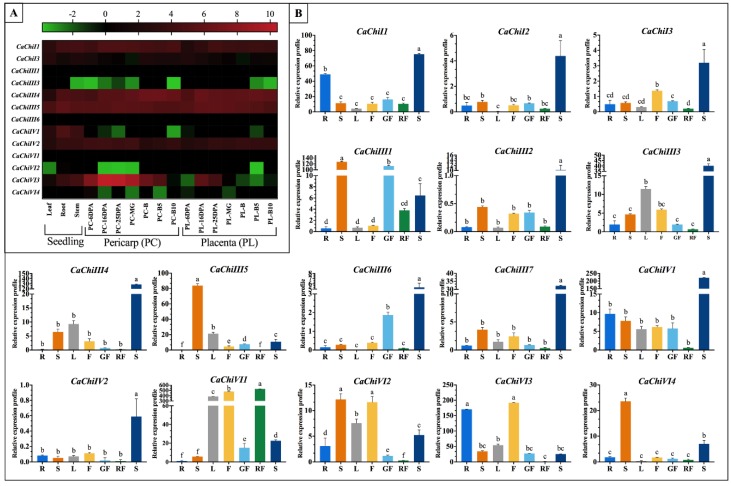
Developmental expression profile of chitin-binding protein family gene in pepper. (**A**) The expression pattern retrieved from the database of pepper (CM334), indicating different expression levels of CaChi genes in dissimilar organs. The results were log2 transformed before generating heat maps in leaf, root, stem, 6, 16, 25 days post-anthesis (6DPA, 16DPA, and 25DPA), mature green (MG), breaker (B), 5 and 10 days post-breaker (B5 and B10) of pericarp (PC) and placenta (PL). Three genes (*CaChiI2*, *CaChiIII2* and *CaChiIII7*) were from the Zunla-1 database, so they were not mentioned in the figure. (**B**) The graphs indicate tissue specific expression levels of chitin-binding protein family genes in pepper plant. The samples were collected from different parts root (R), stem (S), leaf (L), flower (F), green fruit (GF), red fruit (RF) and seed (S) analyzed by qRT-PCR. Data are the means of three independent qRT-PCR amplifications. Small letters (a–f) represent significant differences (*p* < 0.05).

To further elucidate the expression characteristics of CaChi genes in various vegetative and reproductive tissues (leaf, stem, root, seven developmental phases of placenta and pericarp), we carried out in silico analysis using public transcriptomic database of pepper [39,40]. The different expression patterns of CaChi in pepper exhibited higher variance in distinctive tissues and stages, as demonstrated in heat map where from green to red display the index of expression (Figure 10A). Moreover, some CaChi had highest expression levels in all stages of plant growth and development, such as *CaChiI1*, *CaChiIII4*, *CaChiIII5*, *CaChiIV2* and *CaChiVI3*, while some of them had very low or no expression in all the tested tissues, i.e., *CaChiI3*, *CaChiIII1*, *CaChiIII6* and *CaChiVI1*. (Figure 10A). Some genes (*CaChiIV1* and *CaChi13*) are expressed only in particular tissue, mostly in seedling stage. Additionally, to further authenticate the CaChi expression level in various vegetative and reproductive tissues, we cultivated AA3 pepper variety in normal condition and different tissues were collected at different stages. Gene specific primers were used for qRT-PCR analysis (Table S5). As shown in Figure 10B, the expression pattern of CaChi dominantly expressed in seed except *CaChiVI2*, *CaChiVI3*, *CaChiVI4* and *CaChiIII5* expression were maximum in stem/flower, stem and stem, respectively. The lowest expression was recorded in red fruit comparing to other tissues except *CaChiVI1* (529.1), where the particular gene highly correspond to the development of red fruit, flower and leaf. Therefore, it can be assumed that the expression pattern implied by *CaChiVI1* may function importantly in red fruit development. Moreover, *CaChiI2*, *CaChiIII2*, *CaChiIII6*, *CaChiIII7* and *CaChiIV2* did not show highly significant expression to any tested tissues excluding seed but *CaChiIII6* having same results in silico. The integrated investigation of publicly available dataset revealed the ubiquitous expression of these genes and a number of CaChi exhibited a certain degree of tissue specificity. The noticeable differences in both expression level might be related to variation in biological materials, regulation of transcript, interpretation methodology and environmental fluctuation.

### 2.9. Reduced Tolerance of CaChiIV1-Silenced Pepper Plants to NaCl

**Figure 11:**
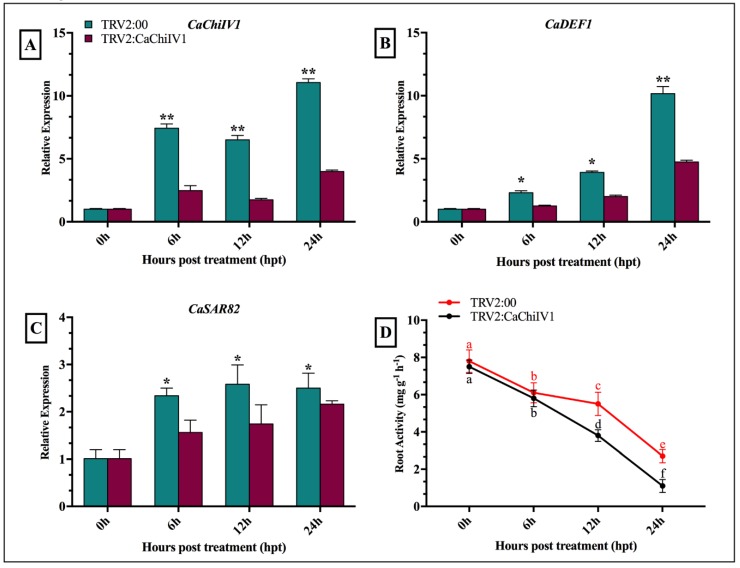
The *CaChiIV1*-silenced pepper plants exhibit reduce resistance to NaCl stress: (**A**) The transcript level of *CaChiIV1*; (**B**) transcript level of *CaDEF1*; (**C**) transcript level of *CaSAR82*; and (**D**) root activity of the control and *CaChiIV1*-silenced plants. Values are the means ± SD from three separate experiments. Small letters (a–f) and asterisk (*significant and **highly significant) denote significant variation (*p* < 0.05).

To evaluate the role of *CaChiIV1* under NaCl stress, the empty vector (used as control) and *CaChiIV1*-silenced plants were treated with NaCl (300 mM) solution. As shown in Figure 11A, the silencing of *CaChiIV1* significantly compromised resistance to NaCl stress. A greater transcript level of *CaChiIV1* was noted in the control (empty vector) plants than in the *CaChiIV1*-silenced plants in all time points, which is >3 folds. In addition, the transcript levels of other defense-related genes were also studied to see whether the silencing of *CaChiIV1* changes their expression. It was noted that, with the passage of time after NaCl treatment, the expression of *CaDEF1* (defensin) [41] and *CaSAR8.2A* (systemic acquired resistance) [42] were changed, but their rise in the control plants (empty vector) were greater compared to *CaChiIV1*-silenced plants (Figure 11B,C). Additionally, root activity was also studied, and the results revealed a significant decrease in the root activity after NaCl stress. At 24 h post NaCl stress, the root activity of the *CaChiIV1*-silenced plants (1.1) was less than TRV2:00 (2.7) (Figure 11D).

## 3. Discussion

Chitin-binding protein (CBP) is an important biotic and abiotic resistance responsive multigene family in plant [20,32,33,43], which play an important role to enhance resistance against stresses in different crops [15]. Chitin-binding proteins are well characterized class of PR proteins [44] which are speculated to be involved in the production of proline due to proline and glycine-rich region [20]. The number of chitin-binding protein genes varies in different plant species. Formerly, 24 chitins in *Arabidopsis thaliana*, 37 in *Oryza sativa* and 17 chitin genes in *Saccharum officinarum* were reported [20,32]. 

In the past, no comprehensive study has been conducted on genome-wide identification and characterization of chitin-binding proteins in pepper. Therefore, in the current study, we retrieved 16 CaChi genes from “CM334” and “Zunla-1” databases of pepper genome. Previously chitins were also found in different plant species [15,20,32,45]. The structural analysis revealed that out of 16 CaChi, eight (50%) CaChi contained introns in which seven genes (43.75%) had only one intron while *CaChiIV1* contain two introns (Figure 3c). Consequently, previously studies on the chitin genes in brassica and banana showed contrasting results [33,46]. The reason may be due to expansion in CaChi family in pepper plant and it may be concluded that the CaChi have undergone diverse gene structure changes during evolution process. The ORFs analysis revealed that the amino acid (aa) sequences ranged from 85 aa (*CaChiVI1*) to 331 aa (*CaChiI1*). The subcellular location of all the CaChi in pepper were predicted, and found that they exist in chloroplast, extracellular region, nucleus, cytoplasmic and vacuolar locations (Table 1). Nishizawa et al. (1999) and Collingel et al. (1993) [47,48] also identified chitins genes in *Oryza sativa*, *Pisum sativum* and *Hordeum vulgare* and described their subcellular localization in vacuole and extracellular.

Previous studies revealed that the nomenclature of the chitins gene family had seven (I–VII) distinct classes [4,15,33]. Therefore, in our study, we also classified CaChi genes with previous criteria into four classes (classes I, III, IV and VI). Parallel, results were also obtained by Backiyarani et al. (2015) [46]. Thus, there is strong possibility that the classification may be due to the homology of the protein sequences and the presence of core conserved domain and probably also in function. The phylogenetic analysis showed that CaChi genes can be divided into four distinct classes: *CaChiI*, *CaChiIII*, *CaChiIV* and *CaChiVI*. It was noticed that sequences contain the glyco_hydro_19 super family, chitinase_glyco_hydro_19 and barwin domains, and they were classified into classes I, IV and VI, respectively (Figure 2). Typical CaChi exhibited higher similarity in the sequence of their conserved domains but an obvious diversity in gene structure and protein size (Figure 1 and Table S2), implying an evolutionary relationship between CaChi genes. It shows that CaChi share a common ancestor and some similar biological functions [32,49].

The chromosomal locations exposed that CaChi were detected on seven diverse chromosomes of *Capsicum annuum*, the highest number of CaChi genes (5) were found on chromosome 3 followed by 7 which had four CaChi. In the duplication and transposition analysis of CaChi, we obtained five clusters of segmental duplication and two genes tandemly duplicated on chromosome 8 (Figure 4). Similarly, our findings are also supported by Cannon et al. (2004) and Backiyarani et al. (2015) [46,50], who also found that tandem and segmental duplication events in *Musa* spp. Therefore, the expansion of a gene family might be segmental and tandem duplication or transposition is the core evolutionary tools [50]. Thus, comparing with tandem duplication and transposition, segmental duplication happens more frequently because of polyploidy in utmost plants, which conserve numerous duplicated chromosomal blocks in their genomes [50]. These conclusions suggest that the pepper CaChi genes endured a complicated evolutionary history during the gene expansion and functional divergence.

Tissue specific expression is a common characteristic of the genes of a certain protein family in plants, which often reflects the functional collaborative and/or differences of the family members [51]. The preceding studies also shed light on developmentally regulated plant chitinases, clarifying their role in the specific physiological processes [52]. To further clarify the possible functions of the CaChi in the growth and development of *Capsicum annuum*, the transcription profiles of CaChi were studied through qRT-qPCR in different tissues. The results showed that mostly CaChi genes exhibited the highest expression during seed formation, followed by the stem, flower, leaf, root and green-fruit (Figure 10B), while the lowest expression was detected in red-fruit. This is similar to previous studies on *Brassica*, where comparatively higher expression levels were observed in flower followed by stem, leaves and roots [33]. Furthermore, Su et al. (2015) [53] also detected chitin gene expression in sugarcane and found high expression in stem pit compared with leaf, and stem epidermis. The transcriptomic analysis as shown in Figure 10A also showed the same expressions in most of the tested tissues but in some case, the transcriptional level is changes may be because of different cultivar was used and some other environmental factors may be involved. The pepper CaChi were expressed in an organ-specific way, suggesting the probable functions in distinct biological processes. CaChi genes suggesting their specific roles in heading stage implying its vital roles in growth regulation and stress response in pepper plant.

Plant diseases activated by fungal pathogens are one of the main concerns and promote defense to a plant pathogen is a difficult mechanism, which includes the triggering of several immune responses [1]. Several resistance genes, including pathogenesis related proteins, have been isolated and were used to improve the defense to different disease in plants. The pathogenesis related proteins are present during hypersensitive response to pathogens of bacteria, virus, fungi and are responsible for the induced resistance in plants. Although it is found that PR proteins not only develop resistance in plants but also play role against pathogen as well as abiotic stresses in susceptible condition [54,55]. In nature, the PR proteins are widely found in the form of chitins genes and play crucial role in plant defense system against the pathogen attack. Multiple antifungal chitinases (CH1, CH2 and CH3) have been reported in various crops such as, from *Sorghum bicolor* [15,56]. Recently, wheat class VII chitinases showed broad-spectrum antifungal activity against *Alternaria* sp., *Sarocladium oryzae*, *Fusarium* *sp.*, *C. falcatum*, *Pestalotia theae* and *Rhizoctonia solani*. The partial mRNA sequences of the chitins *ScChiB1* were amplified from both cultivars (red rot-compatible and incompatible) of sugarcane [57]. In addition, chitin family genes were found to be involved in proline synthesis and primarily associated with defense and resistance against pathogens [32,49], and Western blotting study revealed greater and faster accumulation of chitin genes in a red rot-resistant cultivar [58].

The cellular predicted function of CaChi proteins indicated that they had mainly involved in defense response to fungi, bacterium infection, cell wall macromolecule catabolic process, chitin catabolic process and also in other stresses. The molecular functions of chitinase activities and chitin binding were predicted during GO analysis (Figure 6). Our results correlate with the results of Kovacs et al. (2013) and Rahul et al. (2013) [21,57], who find the functions of chitins genes in *Musa* spp. and *Saccharum officinarum*, respectively. In the current research work, during *Phytophthora capsici* inoculation (0–7 hpi), the transcription level of at least 11 CaChi (*CaChiI2*, *CaChiI3*, *CaChiIII1*, *CaChiIII2*, *CaChiIII4*, *CaChiIII6*, *CaChiIII7*, *CaChiIV1*, *CaChiVI1*, *CaChiVI2* and *CaChiVI3*) were highly induced by both strains (Figure 7). Moreover, the transcript levels of *CaChiI2*, *CaChiIII4* and *CaChiIII7* were higher in response to the PC-strain, whereas *CaChiI3*, *CaChiIII2*, *CaChiIV1*, *CaChiIV2*, *CaChiVI2* and *CaChiVI3* showed higher expression after inoculation with the HX-9 strain versus the PC-strain. These results suggest that CaChi genes are pathogen-inducible and perhaps also participate in immune system of pepper plant, thus helping in disease resistance. It has been found that CaChi are responsive to several biotic stresses and their transcripts were greatly increased (Figure 7). An earlier study also showed that chitins genes were induced by a fungus via *Phytophthora capsici* [49], whereas a class III sugarcane chitinase gene (*ScChi*) was exposed to be induced by *S. scitamineum* [59]. The expression of the *CABPR1* gene in pepper was higher in the virulent strain interaction versus the avirulent interaction [60]. However, in contrast to our previous studies [61], it was found that the expression of most *CaSBPs* was comparatively greater in the avirulent interaction than in the compatible interaction. Some other studies have revealed that the expression of oxysterol-binding protein gene (*CanOBP*) and a novel peroxidase gene (*CanPOD*) were higher in the incompatible interaction [62,63] and the defense-related genes such as b-1, 3-glucanase gene (*CABGLU*), disease-associated protein gene (*CABPR1*), and peroxidase gene (*CAPO1*) were expressed in a similar pattern, after the inoculation of virulent and avirulent strains of *Phytophthora capsici* on the roots of pepper as reported by Wang et al. (2013) [62]. The differences in the expression patterns of CaChi and other defense-related genes might be because of dissimilarities in the inoculation of *Phytophthora capsici* strains, duration of infection, cultivar or the variation in their compatibility systems. 

Earlier studies have shown that chitins genes were expressed in different patterns in response to various abiotic stresses [16,59,64]. Based on GO analysis, CaChi genes imply a role in different stresses, in support of our study. Kumar et al. (2017) [65] also investigated the *OsWRKY71* gene enhanced cold tolerance and mainly involved in metabolic as well as in regulatory pathways in rice, while Eroglu and Aksoy (2017) [66] studied COP9 response under Fe deficiency in Arabidopsis. Hence, we extended our study to investigate the expression analysis of eight representative CaChi after salt (NaCl), cold (6 °C) and drought (mannitol) stresses (Figure 8). The results revealed that *CaChiI3*, *CaChiIII2*, and *CaChiIVI* genes showed significant response to mannitol, while *CaChiIII4*, *CaChiIII7*, *CaChiIVI CaChiIV2* and *CaChiV12* exhibited greater response to cold stress. Therefore, we assumed that chilling induced intracellular Ca^2+^ overload may enhance the ROS production which is key component response to chilling stress. The *CaChiIII6*, *CaChiIII7* and *CaChiIVI* genes expression was maximum at 6 hpt salt stress treatment. These results are supported by Yin et al. (2014) [67] who studied *CaAQP* gene in pepper under salt stress and conform the highest expression at 4 h salt stress but in latter hours the expression was dramatically reduced. The reason for downregulation of permeability of membrane is it results in limitation of water loss from vacuole. These effects propose that the dissimilar pepper chitin-binding protein have separate functions in response to numerous environmental stresses. However, as discussed above, different CaChi exhibit strong spatiotemporal and tissue-specific expressions, demonstrating an obvious collaborative and/or divergence in both biological roles and evolutionary relationship of the chitin-binding protein family genes in pepper.

Chitin-binding protein in plants are responsive for the certain level of abiotic (low temperature, drought, heavy metals and salt) stresses and plant hormones [1,58,59]. Previous reports show that MeJA. SA and ethylene are involved in signal compounds inducing two kinds of defense such as for induced systemic resistance (ISR) and systemic acquired resistance (SAR) [68]. The basic defense to biotrophic pathogens is mediated by SA [3]. In plant responses to environmental cues, MeJA plays the fastest role in resistance reaction via signal molecule reaction center and the genes which are related to MeJA showed upregulation, causing hyper accumulation of MeJA under biotic and abiotic stresses [69]. Several hormonal responsive elements were located in the promoter regions of CaChi, e.g. for MeJA (CGTCA-motif) [70] and SA (TCA-element) [71]. In light of this evidence, pepper plants were exposed to SA and MeJA stresses and their effects on the expression levels were investigated. The expression pattern of CaChi could be differentially regulated by MeJA, ABA and SA (Figure 9). External application of SA lead in an increased accumulation of *CaChiI1*, *CaChiIII2*, *CaChiIII6*, *CaChiIV1*, *CaChiIV2* and *CaChiIV2* expression, *CaChiIII7*, *CaChiIV1* and *CaChiVI2* showed maximum expression level against MeJA and *CaChiIII2*, *CaChiIII6*, *CaChiIII7* and *CaChiV12* exhibit increased transcription by ABA application. It should be noticed that the expression profiles of the members of CaChi have distinct characteristics approach in response to these hormone treatments. For example, Guo et al. (2013) [72] reported that the ABA responsive genes in pepper reduce cold stress injuries and help plants to combat unfavorable environment. Similarly, the evaluated level of these hormones may enhance the antioxidant activity also reduce the accumulation of reactive oxygen species (ROS) and thus play a crucial role in signaling pathways and ultimately in plant defense system.

**Figure 12:**
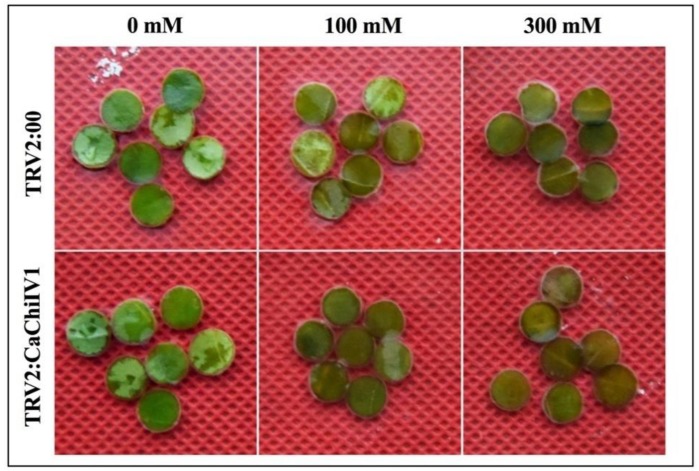
The *CaChiIV1*-silenced pepper plants reduced resistance to NaCl stress. Leaf discs phenotypes (0.5 cm in diameter) of the *TRV2:CaChiIV1* and TRV:00 plants in response to 0 mM, 100 mM and 300 mM NaCl stress after 48 h.

For functional characterization of *CaChiIV1* gene initially, we searched the dataset of *cis-*regulatory elements in the promoter region and then we successfully knocked them down in pepper plant. Further, we performed an expression analysis under salt stress condition (Figure 11A). The results displayed significant response to salt stress as well as the defense related gene when compared to control plants. Similar to our results, rice *OsDIRs* exhibit greater response to salt stress as compared with mock-treated control seedlings [73]. Wu et al. (2009) [74] also reported similar finding during their studies on dirigent protein gene from the resurrection plant *Boea hygrometrica*. In current study, we also performed the detached leaf assay of the *CaChiIV1*-silenced gene with control plants treated with salt stress. The silenced plants showed the reduction in chlorophyll content, suggesting that due to knock down of *CaChiIV1* gene the pepper leaves are more susceptible to the NaCl stress (Figure 12). In pepper, the dehydrin *CaDHN1* silenced plants showed decrease in chlorophyll contents after three days of salt treatments [75]. Thus, the degree of leaf senescence in *CaChiIV1*-silenced plants are greater than control. Furthermore, the TTC reductase activity in the roots of pepper plant was measured after NaCl stress in both silenced and control plant with a duration of 0–24 h. the significant reduction in root activity was seen in silenced than control plants (Figure 11D). The root activity was significantly reduced of the silenced plants in avirulent strain of *P. capsica* than virulent strain [62]. Moreover, the *CaPTI1* gene in pepper also showed the significant differences in root activity [76]. The reason plants are susceptible to salt stress is that it causes severe injury in root tips and may lead to reduction in root activity. In conclusion, the *CaChiIV1* gene demonstrated a crucial role in biological processes and functionally involved in NaCl stress and defense response in pepper plant.

## 4. Materials and Methods

### 4.1. Identification and Sequence Analysis of CaChi Genes Family in Pepper

To identify the CaChi family members based on the conserved domain, accession No. “PF00187.17” was collected from Pfam database (http://pfam.xfam.org/) as described in our previous study [61,77]. To further authenticate the CaChi family members, the CaChi were aligned with DNAMAN to cross check in both CM334 (Available online: http://peppergenome.snu.ac.kr/download.php) [40] and Zunla-1 (Available online: http://peppersequence.genomics.cn/) [78] databases of pepper, while the obtained sequences were from the latest versions, i.e., v1.55 and v2.0, respectively. Furthermore, gene-specific primer pairs (Table S1) were designed by using Primer Premier 6.0 (Premier Biosoft International, Redwood City, CA, USA) to amplify the different target regions.

### 4.2. Phylogenetic Relationships, Sequence Alignment and Physio-Chemical Properties of CaChi Genes 

Multiple sequence alignment of the pepper chitin-binding proteins were performed by ClustalW according to previous studies on plant chitinases [15,32,33]. The phylogenetic tree was built with iTOL (Available online: https://itol.embl.de/) [79] using neighbor-joining (NJ) method with 1000 bootstrap replicates. Nomenclature of the putative CaChi genes were assigned based on their class and chromosomal order. To compute the molecular formula, total number of items, instability index, molecular weight (MW), molecular formula (MF) and theoretical isoelectric point (*p*I), the amino acid sequences were blast in Expasy ProtoParam (Available online: http://web.expasy.org/protparam/) [80] and WoLF 32 PSORT II (Available online: http://www.genscript.com/wolf-psort.html) [81]. The TargetP online tool (Available online: http://www.cbs.dtu.dk/services/TargetP/) [82] was used to predict the subcellular locations.

### 4.3. Exon–Intron Structure Analysis, Conserved Motifs and Domain Architecture

The gene structures (exon–intron) of CaChi were obtained by aligning the CDS sequences with their corresponding genomic sequences, and their structures were shaped by using online Gene-Structure-Display Server 2.0 as described by Kang et al. (2016) [83]. Conserved motifs of the CaChi genes were recognized using MEME tool (4.12.0) (Available online: http://meme-suite.org/tools/meme) as described by Guo et al. (2016) [84] with maximum number of motifs = 10. To increase the confidence level, all candidate protein sequences were further scrutinized for presence of the functional domains by the online tools, Conserved Domain Database (CDD) (Available online: http://www.ncbi.nlm.nih.gov/cdd/), SMART (Available online: http://smart.embl-heidelberg.de/), and EMBL-EBI (Available online: https://www.ebi.ac.uk/interpro/), and their diagrams were generated using online EXPASY server (Available online: https://prosite.expasy.org/mydomains/).

### 4.4. Analysis of Cis-Regulatory Elements and Gene Ontology of CaChi

To analyze the *cis*-acting elements, 1500 bp upstream from the start codon (ATG) of the CaChi were obtained from the pepper genome database (PGD) and queried against the PlantCARE (Available online: http://bioinformatics.psb.ugent.be/webtools/plantcare/html/) [85] and Neural Network Promoter Prediction (Available online: http://promotor.biosino.org/) online servers. The gene ontology analysis of CaChi protein sequences were obtained from Blast2GO (Available online: http://www.blast2go.com) program [86].

The amino acid sequences were blast in Blast2GO program and three groups of GO classification (molecular functions, biological process and cellular component) were recovered.

### 4.5. Chromosomal Location and Genes-Duplication Analysis of CaChi Genes

Information about the chromosomal location of CaChi genes were obtained from Pepper Genome Platform (PGP) (Available online: http://peppergenome.snu.ac.kr/) as described by Zhang et al. (2016) [61], and the genes were mapped on chromosomes using MapDraw [87]. Duplication analysis within the pepper genome was carried out with the criteria depicted by Gu et al. (2002) [88] further detail as: (1) the FASTA-alignable region among the two proteins should be more than 80% of the longer protein sequence; and (2) the identity between the two protein sequences (I) should be I ≥ 30% if the alignable region is longer than 150 amino acid and I ≥ 0.01*n* + 4.8L^−0.32(1 + exp(−L/1000)^ otherwise, where *n* = 6 and L is the alignable length between the two protein sequences of the gene [88,89].

### 4.6. Transcriptomic Data Analysis of the CaChi in Different Tissues

Publicly available transcriptomic data of root, stem, leaf, and for both pericarp and placenta at mature green (MG), breaker (B), 5 days post-breaker (5B), 10 days post-breaker, 6 days post anthesis (6DPA), 16 days post anthesis (16DPA) and 25 days post anthesis (25DPA) for pepper cultivar CM334 were retrieved from online server (Available online: http://peppergenome.snu.ac.kr/) which have been generated previously by Kim et al. (2014) [40]. The data were based on the Reads Per Kilobase per Million mapped reads (RPKM) analysis indicating the transcriptomic level of CaChi members and the results were presented using a heat map.

### 4.7. Plant Materials and Inoculation with Phytophthora capsici Strains

Pepper cultivar AA3, as well as *P. capsici* strains were obtained from the Laboratory of Vegetable Plant Biotechnology and Germplasm Innovation, Northwest A&F University-China. The method used for *P. capsici* inoculation was same as described by Khan et al. (2018) and Zhang et al. (2016) [61,77]. The root samples were amassed 0, 6, 12, 24, 48 and 72 h post inoculation (hpi) of *P. capsici*. For tissue specific expression, samples were collected from roots, stems, leaves, flower, green fruits, red fruits and seeds of untreated pepper plant for RNA extraction and qRT-PCR analysis [76,77].

### 4.8. Hormonal Applications and Abiotic Stresses Treatments

For hormonal treatments the plantlets were treated with 50 μM methyl jasmonate (MeJA), 5 mM salicylic acid (SA) and 0.57 mM ABA solution [90]. Plantlets were kept at 28 °C in condition of 16 h light/8 h dark photoperiod and samples were collected at 0, 1, 3, 6, 12, 24 and 48 h post treatment (hpt). For abiotic stresses, some pepper seedlings were exposed to low temperature (6 °C) in chamber and others separately treated with 300 mM both NaCl and mannitol for 0, 1, 3, 6, 12, 24 and 48 hpt [72]. The samples were directly frozen in liquid nitrogen after harvesting and saved at −80 °C for RNA extraction. The experiments were carried out in three biological replicates.

### 4.9. RNA Extraction and qRT-PCR Analysis

Total-RNA was extracted from different samples using Trizol reagent (Invitrogen, Carlsbad, CA, USA) following the instruction of manufacturer’s protocol. Further treatment of RNA was achieved with RNase-free DNaseI to eliminate DNA contamination. The cDNA was synthesis by using the Prime-Script TM RT Reagent Kit (TaKaRa, Dalian, China). The quality of cDNA was checked by nanodrop (Thermo Scientific NanoDrop 2000C, Wilmington, DE, USA) and the required volume was calculated and adjusted the concentration up to 50 ng/µL. For qRT-PCR analysis, the gene-specific primers (Table S5) were designed by using Primer Premier 6.0 software package (Available online: http://www.premierbiosoft.com/primerdesign/index.html). The specificities of the primers were further confirmed through NCBI Primer BLAST (Available online: https://www.ncbi.nlm.nih.gov/tools/primer-blast/). The pepper ubiquitin-conjugating protein gene (*CaUbi3*) was used as internal control [91], with a little modification of annealing temperature (60 °C for 30 s). The relative expression levels of all the CaChi genes were calculated using the 2^−ΔΔCt^ method [92].

### 4.10. VIGS Assay of CaChiIV1

The VIGS approach was used for the knock-down of the *CaChiIV1* gene in the pepper plant cultivar AA3, and the VIGS assay was performed as described by Liu et al. (2016) [93]. For *pTRV2:CaChiIV1*, a 232-bp cDNA part of *CaChiIV1* gene was PCR-amplified. Briefly, the *CaChiIV1* gene was cloned into a pTRV2 vector to construct the recombinant plasmid *pTRV2:CaChiIV1*, which was further used in the subsequent research to confirm the exact silencing of *CaChiIV1* (primer pairs used for vector construction are given in Table S5. Afterwards, the freeze–thaw method was used to transform pTRV1, pTRV2 (negative control), and *pTRV2:CaPDS* (positive control) along with the combined vector *pTRV2:CaChiIV1* into an *Agrobacterium tumefaciens* strain (GV3101). *A. tumefaciens* harboring pTRV1 was mixed at a 1:1 ratio with pTRV2, pTRV2-CaPDS and pTRV2-*CaChiIV1*. The agrobacterium inocula suspensions harboring pTRV1, pTRV2:00, *pTRV2:CaPDS* or *pTRV2:CaChiIV1* (OD600 = 1.0) were infiltrated into the full extended cotyledons leaves of pepper plants using a 1.0 mL clean needleless syringe [94]. Then, these infiltrated plants were conserved at 18–22 °C in a plant growth chamber with a 16/8 h light/dark period as defined by Wang et al. (2013) [62,95]. Forty-five days post-infiltration, leaf samples from the control and *CaChiIV1*-silenced plants were collected to measure the silencing efficiency by RT-PCR. The triphenyltetrazolium chloride (TTC) method was used to measure the root activity [62,95]. Before the TTC test, root tips (approximately 0.2 g) from the control (TRV:00) and *CaChiIV1*-silenced (*pTRV2*:*CaChiIV1*) plants were collected at various time points after NaCl stress as described by Khan et al. (2018) [77]. These experiments were executed with three biological repeats.

### 4.11. Statistical analysis

The results were subjected to an analysis of variance (ANOVA) using SPSS software (SPSS version 23.0, SPSS Inc., Chicago, IL, USA), and the analyzed data were expressed as means ± standard deviation (SD) of three replications in all measured parameters. The least significant difference (LSD; *p* < 0.05) test was used to measure the significant differences among the given treatments. Data were presented in graphs and designed using GraphPad Prism 7.0 (GraphPad Software, Inc., LA Jolla, CA, USA).

## 5. Conclusions

The findings of our research work briefly explain that the pepper chitin-binding protein gene family performs a vital role in the complex signaling networks system in response to numerous biotic and abiotic stresses and exogenous hormone applications. Every member of CaChi has its specific expression pattern and functional preference. Meanwhile, the particular molecular mechanism and function of pepper chitin-binding protein are still unclear. Additionally, the chitins–genes connections are also weakly discussed. The various transcription pattern of pepper chitin genes has been observed in tissues due to fluctuations in environmental circumstances, for example salt, drought and hormones, suggesting the diverse roles and inimitable transcription levels of chitin genes in the pepper plant growth, development and response to different stresses. Accordingly, different changes in transcription level in the same pepper chitin gene against several biotic and ambient alterations confirm the differences in their mechanism of regulation. These outcomes clarify the background for further experiments and provide the basic knowledge to explore the role and the possible cross-talk between pepper chitin-binding proteins in plants.

## Figures and Tables

**Table 1:** List of Chitin-binding protein family genes identified in pepper and their sequence characteristics. Chr: chromosome; CDS: codding sequence; MW: molecular weight (kDa). the proteomic information was obtained from ExPASy (Available online: http://web.expasy.org/protparam/).

Name	Gene Locus ID	Chr	Position	CDS (bp)	MW	Instability Index	Introns
*CaChiI1*	*Capana07g001653*	7	195532737–195534304	996	35.49	38.08	2
*CaChiI2*	*Capana10g001143*	10	114626443–114627269	564	20.41	39.74	1
*CaChiI3*	*CA10g09850*	10	146386994–146387462	468	16.45	40.99	0
*CaChiIII1*	*Capana03g000778*	3	11663598–11664075	477	17.09	51.20	0
*CaChiIII2*	*Capana03g000780*	3	11754372–11754972	600	21.30	58.21	1
*CaChiIII3*	*CA03g30170*	3	245663113–245663923	810	28.93	55.61	0
*CaChiIII4*	*CA03g30180*	3	245700488–245700986	498	17.80	68.89	0
*CaChiIII5*	*CA03g30190*	3	245839016–245839764	618	22.45	35.35	1
*CaChiIII6*	*Capana07g001180*	7	161369376–161369835	459	16.15	47.70	0
*CaChiIII7*	*Capana07g001181*	7	161402785–161403394	609	21.38	64.04	0
*CaChiIV1*	*CA00g54030*	4	193080176–193081152	834	30.06	18.45	1
*CaChiIV2*	*Capana06g002084*	6	87334514–87335721	759	27.93	30.88	1
*CaChiVI1*	*CA07g09480*	7	147837320–147837578	258	9.06	22.23	0
*CaChiVI2*	*Capana08g001237*	8	126990120–126991323	606	21.31	21.57	1
*CaChiVI3*	*CA08g10220*	8	128199078–128200438	627	22.37	26.39	1
*CaChiVI4*	*CA12g08860*	12	49994036–49994360	324	11.94	34.02	0

## Supplementary Materials

Supplementary materials can be found at http://www.mdpi.com/1422-0067/19/8/2216/s1.
