# Supplementary material for: Classification and Genome-Wide Analysis of Chitin-Binding Proteins Gene Family in Pepper (Capsicum annuum L.) and Transcriptional Regulation to Phytophthora capsici, Abiotic Stresses and Hormonal Applications
Source: Int J Mol Sci. 2018 Jul 29;19(8):2216. doi: 10.3390/ijms19082216 (PMC6121964; doi:10.3390/ijms19082216)
Supplement: Supplementary file 1 [file ijms-19-02216-s001.pdf]

Classification and genome-wide analysis of chitin-binding proteins gene family in pepper (*Capsicum annuum* L.) and transcriptional regulation to *Phytophthora capsici*, abiotic stresses and hormonal applications

Muhammad Ali, De-Xu Luo, Abid Khan, Saeed ul Haq, Wen-Xian Gai, Huai-Xia Zhang, Guo-Xin Cheng, Izhar Muhammad, Zhen-Hui Gong

Table S1. Primers for gene sequencing and confirmation.

Gene Name	Accession#		Primer sequence (5'→3')
CaChiI1	Capana07g001653	F	ATGAAGCTTTGTGAATTCACAGC
		R	TTACATAGAATCGATTAAGAGTCCA
CaChiI2	Capana10g001143	F	CACTTTGTCACTTTCTTTCCATCAC
		R	CCTCGTCAAAAATATTTTCTTCCAG
CaChiI3	CA10g09850	F	GCCACTTGTTTCCCATTACC
		R	CTGATCTCAAAGTTGAACTTATC
CaChiIII1	Capana03g000778	F	ATGATGAGAACTAGAGAGACAGC
		R	CTAGAGAGCATTGAGCAGGAAG
CaChiIII3	CA03g30170	F	CCATCCTGTCATCACAAAATGATG
		R	GGAACGACCAACATCTAGCTAG
CaChiIII5	CA03g30190	F	CGTCTTATAACAATTCACAAAATG
		R	CTTGTTATACGAGGTTAGACC
CaChiIV2	Capana06g002084	F	ATGTTGGCTCAGAATTGTGAGTG
		R	CTAACAAGAGAGATTATCCCCAG
CaChiVI1	CA07g09480	F	GTAAGAAAGAAGCATAAAGAG
		R	GTATCATACGTTCATTAGTTGG
CaChiVI2	Capana08g001237	F	ATGGAGAAGCTAAGTACTACTGC
		R	TCAGTTATCACCACAATCAAC
CaChiVI3	CA08g10220	F	CAACCACATTATAGCAGCTCT
		R	CAGACTAAACAGAGCCATG

Table S2. Chemistry and domains organization of CaChi´s genes of pepper.

Genes	Domains Interval				Formula	Total number of atoms
	Chitin_bind_1	Glyco_hydro_19 super family	chitinase_glyco_hydro_19	Barwin		
CaChiI1	24-62	84-316	---	---	$C_{1554}H_{2366}N_{452}O_{461}S_{23}$	4856
CaChiI2	24-62	84-187	---	---	$C_{897}H_{1375}N_{261}O_{259}S_{14}$	2806
CaChiI3	24-62	84-154	---	---	$C_{715}H_{1096}N_{200}O_{221}S_{13}$	2245
CaChiIII1	47-85<br>97-136	---	---	---	$C_{735}H_{1149}N_{209}O_{223}S_{19}$	2335
CaChiIII2	35-73<br>83-119<br>143-181	---	---	---	$C_{908}H_{1373}N_{271}O_{274}S_{26}$	2852
CaChiIII3	48-86<br>102-140<br>156-194<br>210-248	---	---	---	$C_{1252}H_{1927}N_{349}O_{369}S_{36}$	3933
CaChiIII4	2-36<br>52-90<br>106-143	---	---	---	$C_{756}H_{1151}N_{213}O_{234}S_{26}$	2380
CaChiIII5	57-95<br>103-119	---	---	---	$C_{985}H_{1570}N_{290}O_{279}S_{16}$	3140
CaChiIII6	49-87<br>96-132	---	---	---	$C_{691}H_{1079}N_{187}O_{223}S_{18}$	2198
CaChiIII7	48-85<br>94-132<br>143-181	---	---	---	$C_{894}H_{1400}N_{250}O_{306}S_{26}$	2876
CaChiIV1	33-59	---	78-277	---	$C_{1320}H_{1959}N_{357}O_{414}S_{19}$	4069

CaChiIV2	3-34	---	53-252	---	$C_{1226}H_{1832}N_{330}O_{379}S_{21}$	3788
CaChiVI1	22-59	---	---	---	$C_{374}H_{599}N_{117}O_{127}S_{9}$	1226
CaChiVI2	26-65	---	---	83-198	$C_{913}H_{1423}N_{273}O_{288}S_{15}$	2912
CaChiVI3	26-65	---	---	78-193	$C_{956}H_{1496}N_{288}O_{302}S_{16}$	3058
CaChiVI4	43-80	---	---	---	$C_{514}H_{812}N_{150}O_{152}S_{13}$	1641

Table S3. Ten highly conserved motifs found in CaChi´s proteins.

No.	Motif consensus sequence	E-value	Regular expression (RE) describing the motif.	Length (AA)
1	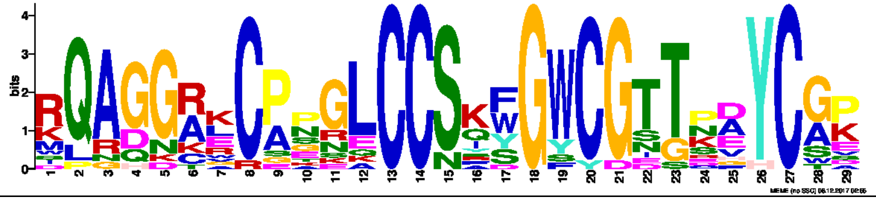	1.5e-156	RQAGGRKCPPGLCCSKFGWCGTTPAYCGP	29
2	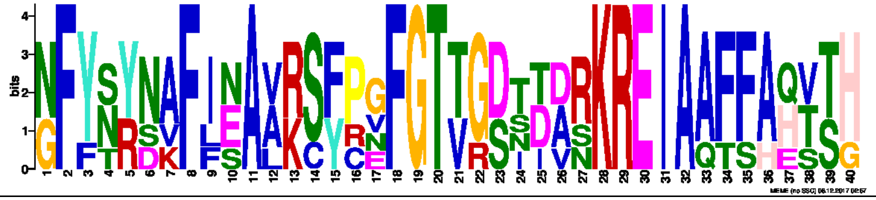	6.8e-060	NFYSYNAFINAVRSFPGFGTTGDTTARKRE IAAFFAQTTH	40
3	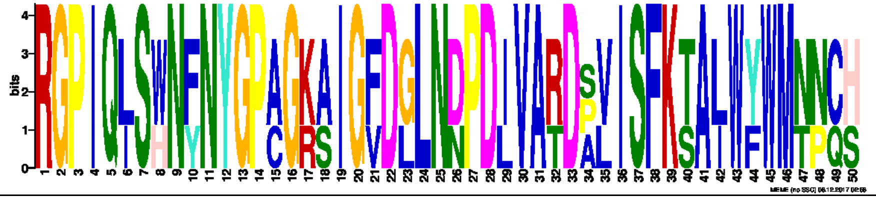	1.2e-044	RGPIQLSWNFNYGPAGKAIGFDGLNDPDIV ARDPVISFKTALWYWMNNCH	50

4	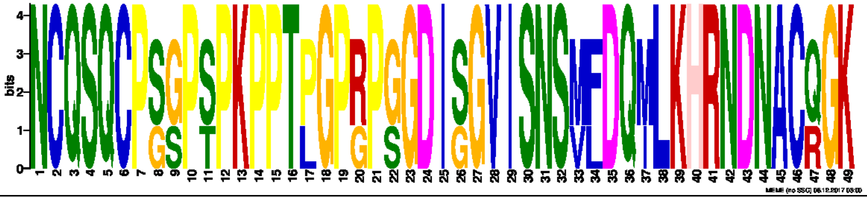	1.2e-039	NCQSQCPSGPSPKPPTPGPRPGGDISGVIS NSMFDQMLKHRNDNACQGK	49
5	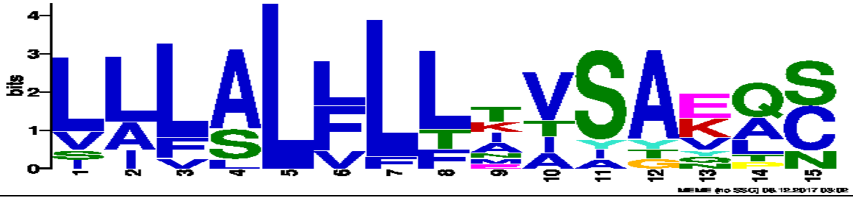	1.6e-034	LLLALLLLTVSAEQS	15
6	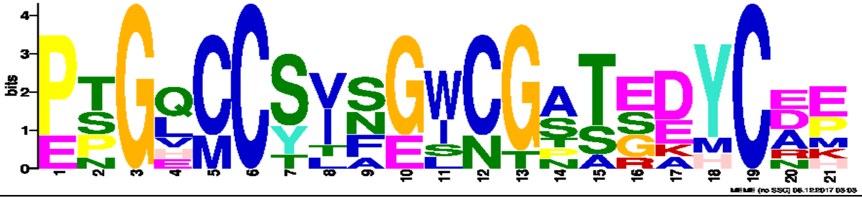	2.2e-024	PTGQCCSISGWCGATEDYCEE	21
7	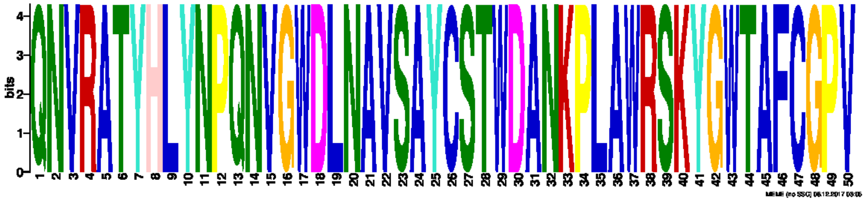	3.8e-023	QNVRATYHLYNPQNVGWDLNAVSAYCSTWD ANKPLAWRSKYGWTAFCGPV	50
8	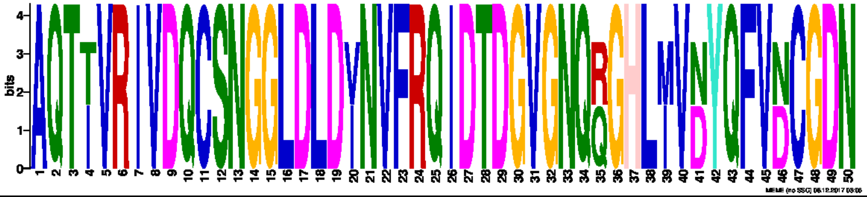	2.2e-019	AQTIVRIVDQCSNGGLDLDINVFRQIDTDGV GNQRGHLIVDYQFVDCGDN	50

9	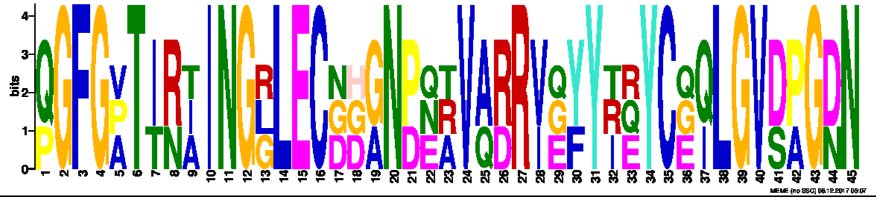	7.6e-017	QGFGPTIRAINGRLECDDGNPQAVARRVEY<br>YTQYCEQLGVDPGDN	45
10	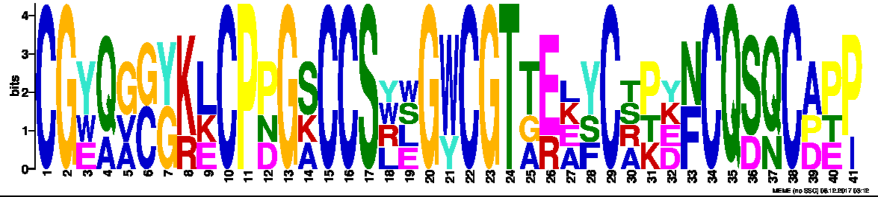	3.3e-016	CGYQGGGKLCPPGSCCSWWGWCGTTEEYCS<br>PEFCQSQCAPP	41

Table S4. Function of the *cis*-acting elements found in the promoter region of CaChi´s.

Cis acting elements	Motif	Function	Reference
Defense-related and stress stimulative elements	TC-rich repeats	Defense and stress responsiveness	[1]
	W box	Fungal elicitor-responsive element	[2]
	WUN-motif	Wound-responsive element	[3]
	HSE	Heat stress responsiveness	[3]
	LTR	Low temperature responsiveness	[4]
	MBS	MYB binding site involved in drought-inducibility	[5]
Plant hormones responsive elements	CGTCA-motif	MeJA responsiveness	[6]
	GARE-motif	GA-responsive element	[7]
	TCA-element	Salicylic acid responsiveness	[8]
	TGA-element	Auxin-responsive element	[3]
	ERE	Ethylene-responsive element	[9]
	ABRE	ABA responsiveness	[10]
	AuxRR-core	Auxin responsiveness	[11]
	P-box	GA-responsive element	[12]

Table S5. Primers for qRT-PCR of CaChi´s genes of pepper.

Gene name	RT-PCR Primer sequence (5'→3')	Product length
CaChiI1	F: AAACCTCCCATGAAACTACCG	227
	R: GTTTAAAAGGTCAACTCCGATGG	
CaChiI2	F: GGACATCAGCGGTGTAATCT	116
	R: CAGCATTGATGAAGGCATTGT	
CaChiI3	F: CTATTTGCTCTCTTATTGCTGATGG	133
	R: CAGGACCACAATAGGCATCG	
CaChiIII1	F: TCTTCCCTTTCATCTTCCAACC	229
	R: AGTTTGTTCTCCGCATCGTC	
CaChiIII2	F: CTCCACCTCCACCTAATTTCC	100
	R: CCACACCAACCCCATCTAC	
CaChiIII3	F: TGGTGCGGAACTACAGAAAAG	127
	R: TTCTACCATCAGCTTGCCTTC	
CaChiIII4	F: GGAATGCAAGCTGATGGTAGA	110
	R: CTCTGACTTTGACACCACTCTT	
CaChiIII5	F: GAAAGCAAGCTGCTGGTAGA	106
	R: TGACTTTGACAGTAGGGAGAAC	
CaChiIII6	F: GTCCTAGTGAAGAGTGTTGTAGTT	114
	R: CGCATAAACCTGGCTCTTCT	
CaChiIII7	F: TCAGCCAAAACATCTCTTCCC	121

	R: CATCAGGGCATTCTCTACCAC	
CaChiIV1	F: TCTTTGCTCATGTCACCCAC<br>R: ATCTCTTGCAACTATGTCAGGG	227
CaChiIV2	F: TCAAGTGCTATCCTGAATTCGG<br>R: CTTTCCAGAGACACAAGGGTAC	182
CaChiVI1	F: CAATACGGGTTCTGTGGTACG<br>R: CAACATTTTCAGCTTCGCCAG	103
CaChiVI2	F: TGGGACTTGAATGCGGTTAG<br>R: TCACTATCGTCTGAGCTCCTG	178
CaChiVI3	F: CGACATGGGATGCTAATAAGCC<br>R: CGTTGTTTGAGCTCTGGTTCG	143
CaChiVI4	F: GTCTTTGTGATTTTGGCCCTG<br>R: TTGGCGAACATGGTAGTGG	191

References

1. Diaz-De-Leon, F.; Klotz, K. L.; Lagrimini, L. M. Nucleotide sequence of the tobacco (Nicotiana tabacum) anionic peroxidase gene. *Plant Physiol.* **1993**, *101*, 1117–1118, doi:10.1104/pp.101.3.1117.
2. Rushton, P. J.; Torres, J. T.; Parniske, M.; Wernert, P.; Hahlbrock, K.; Somssich, I. E. Interaction of elicitor-induced DNA-binding proteins with elicitor response elements in the promoters of parsley PR1 genes. *EMBO J.* **1996**, *15*, 5690–5700, doi:10.1002/J.1460-2075.1996.TB00953.X.
3. Pastuglia, M.; Roby, D.; Dumas, C.; Cock, J. M. Rapid induction by wounding and bacterial infection of an S gene family receptor-like kinase gene in Brassica oleracea. *Plant Cell* **1997**, *9*, 49–60.
4. White, A. J.; Dunn, M. A.; Brown, K.; Hughes, M. A. Comparative analysis of genomic sequence and expression of a lipid transfer protein gene family in winter barley. **1994**, *45*, 1885–1892.
5. Nash, J.; Luehrsen, K. R.; Walbot, V. Bronze-2 gene of maize: reconstruction of a wild-type allele and analysis of transcription and splicing. *Plant Cell* **1990**, *2*, 1039–1049, doi:10.1105/tpc.2.11.1039.
6. Rouster, J.; Leah, R.; Mundy, J.; Cameron-Mills, V. Identification of a methyl jasmonate-responsive region in the promoter of a lipoxygenase 1 gene expressed in barley grain. *Plant J.* **1997**, *11*, 513–523, doi:10.1046/j.1365-313X.1997.11030513.x.
7. Skriver, K.; Olsen, F. L.; Rogers, J. C.; Mundy, J. cis-acting DNA elements responsive to gibberellin and its antagonist abscisic acid. *Proc. Natl. Acad. Sci.* **1991**, *88*, 7266–7270, doi:10.1073/pnas.88.16.7266.
8. Merkouropoulos, G.; Barnett, D. C.; Shirsat, A. H. The Arabidopsis extensin gene is developmentally regulated, is induced by wounding, methyl jasmonate, abscisic and salicylic acid, and codes for a protein with unusual motifs. *Planta* **1999**, *208*, 212–219, doi:10.1007/s004250050552.

9. Itzhaki, H.; Woodson, W. R. Characterization of an ethylene-responsive glutathione S-transferase gene cluster in carnation. *Plant Mol. Biol.* **1993**, *22*, 43–58, doi:10.1007/BF00038994.
10. Baker, S. S.; Wilhelm, K. S.; Thomashow, M. F. The 5′-region of Arabidopsis thaliana cor15a has cis-acting elements that confer cold-, drought- and ABA-regulated gene expression. *Plant Mol. Biol.* **1994**, *24*, 701–713, doi:10.1007/BF00029852.
11. Sakai, T.; Takahashi, Y.; Nagata, T. Analysis of the promoter of the auxin-inducible gene, parC, of tobacco. *Plant Cell Physiol.* **1996**, *37*, 906–913, doi:10.1093/oxfordjournals.pcp.a029038.
12. Kim, J. K.; Cao, J.; Wu, R. Regulation and interaction of multiple protein factors with the proximal promoter regions of a rice high pl α-amylase gene. *MGG Mol. Gen. Genet.* **1992**, *232*, 383–393, doi:10.1007/BF00266241.
